# Supplementary figures and images for: Gut bacterial communities in the freshwater snail Planorbella trivolvis and their modification by a non-herbivorous diet
Source: PeerJ. 2021 Feb 12;9:e10716. doi: 10.7717/peerj.10716 (PMC7883694; doi:10.7717/peerj.10716)

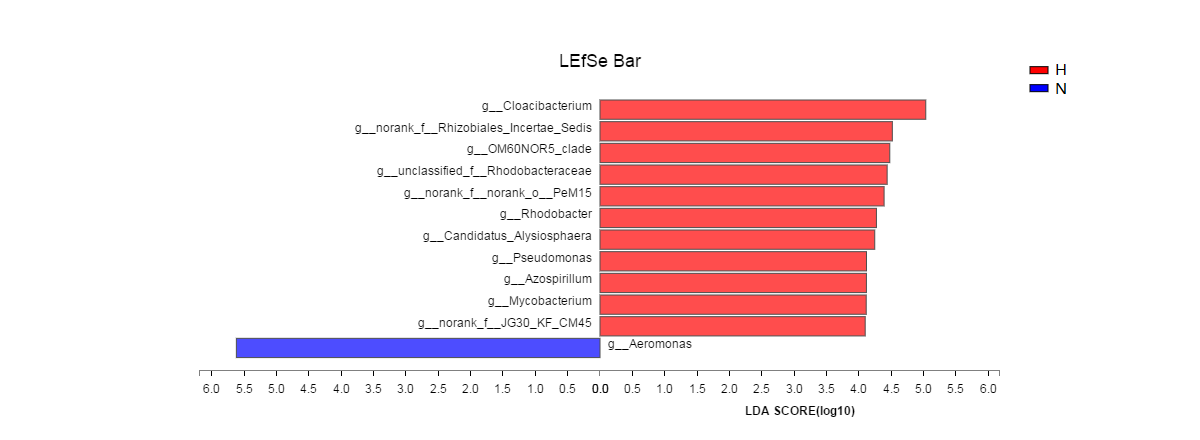

Supplement: Supplemental Information 1 — The HV group is represented by red bars (H) and the NHV group by blue bars (N). [file peerj-09-10716-s001.png]
