# Supplementary material for: Gut bacterial communities in the freshwater snail Planorbella trivolvis and their modification by a non-herbivorous diet
Source: PeerJ. 2021 Feb 12;9:e10716. doi: 10.7717/peerj.10716 (PMC7883694; doi:10.7717/peerj.10716)
Supplement: Supplemental Information 2 [file peerj-09-10716-s002.docx]

| Species name | group | LDA_value | Pvalue |
| --- | --- | --- | --- |
| g__Aeromonas | NHV | 5.62 | 0.01 |
| g__Comamonas | NHV | 3.78 | 0.01 |
| g__Shewanella | NHV | 3.69 | 0.05 |
| g__Haloferula | NHV | 3.46 | 0.05 |
| g__Uliginosibacterium | HV | 3.67 | 0.01 |
| g__Cloacibacterium | HV | 5.04 | 0.01 |
| g__norank_f__Rhizobiales_Incertae_Sedis | HV | 4.52 | 0.01 |
| g__OM60NOR5_clade | HV | 4.48 | 0.01 |
| g__unclassified_f__Rhodobacteraceae | HV | 4.44 | 0.01 |
| g__norank_f__norank_o__PeM15 | HV | 4.39 | 0.01 |
| g__Rhodobacter | HV | 4.27 | 0.01 |
| g__Candidatus_Alysiosphaera | HV | 4.25 | 0.01 |
| g__Pseudomonas | HV | 4.12 | 0.02 |
| g__Azospirillum | HV | 4.12 | 0.01 |
| g__Mycobacterium | HV | 4.12 | 0.01 |
| g__norank_f__JG30_KF_CM45 | HV | 4.1 | 0.01 |
| g__Polymorphobacter | HV | 3.98 | 0.01 |
| g__unclassified_f__Enterobacteriaceae | HV | 3.96 | 0.01 |
| g__Roseobacter_clade_CHAB_I_5_lineage | HV | 3.85 | 0.01 |
| g__Thioclava | HV | 3.85 | 0.01 |
| g__Luteolibacter | HV | 3.8 | 0.02 |
| g__norank _c__Sericytochromatia | HV | 3.77 | 0.01 |
| g__Flavobacterium | HV | 3.77 | 0.01 |
| g__Roseomonas | HV | 3.76 | 0.01 |
| g__Dinghuibacter | HV | 3.75 | 0.01 |
| g__Reyranella | HV | 3.73 | 0.01 |
| g__unclassified_f__Parachlamydiaceae | HV | 3.65 | 0.01 |
| g__Delftia | HV | 3.65 | 0.01 |
| g__norank_f__norank_o__Chloroplast | HV | 3.57 | 0.01 |
| g__Sandaracinobacter | HV | 3.52 | 0.01 |
| g__Polynucleobacter | HV | 3.49 | 0.01 |
| g__Aurantimicrobium | HV | 3.49 | 0.01 |
| g__Terrimicrobium | HV | 3.48 | 0.01 |
| g__Sporichthya | HV | 3.47 | 0.01 |
| g__Bacteroides | HV | 3.46 | 0.01 |
| g__Kluyvera | HV | 3.44 | 0.01 |
| g__norank_f__Chitinophagaceae | HV | 3.38 | 0.01 |
| g__norank_f__Burkholderiaceae | HV | 3.31 | 0.01 |
| g__norank_f__B1_7BS | HV | 3.25 | 0.01 |
| g__Desulfovibrio | HV | 3.24 | 0.01 |
| g__Sphingorhabdus | HV | 3.23 | 0.01 |
| g__Legionella | HV | 3.22 | 0.01 |
| g__IMCC26207 | HV | 3.2 | 0.01 |
| g__Gordonia | HV | 3.11 | 0.02 |
| g__norank_f__norank_o__SJA_28 | HV | 3.08 | 0.01 |
| g__Bdellovibrio | HV | 3.07 | 0.01 |
| g__Microbacterium | HV | 3.05 | 0.04 |
| g__norank_c__Planctomycetacia | HV | 3.04 | 0.01 |
| g__Candidatus_Berkiella | HV | 3.03 | 0.01 |
| g__norank_f__Xanthobacteraceae | HV | 3.03 | 0.01 |
| g__CL500_3 | HV | 2.99 | 0.01 |
| g__Thermomonas | HV | 2.99 | 0.01 |
| g__norank_ o__Gammaproteobacteria_Incertae_Sedis | HV | 2.96 | 0.01 |
| g__unclassified_f__Rickettsiaceae | HV | 2.96 | 0.01 |
| g__Candidatus_Endonucleariobacter | HV | 2.91 | 0.02 |
| g__Leptotrichia | HV | 2.91 | 0.01 |
| g__unclassified_f__Burkholderiaceae | HV | 2.9 | 0.01 |
| g__norank_f__Rickettsiaceae | HV | 2.9 | 0.02 |
| g__Dechloromonas | HV | 2.89 | 0.01 |
| g__norank_f__SC_I_84 | HV | 2.87 | 0.01 |
| g__norank_f__Paracaedibacteraceae | HV | 2.86 | 0.01 |
| g__Aquicella | HV | 2.84 | 0.01 |
| g__norank_f__Barnesiellaceae | HV | 2.79 | 0.01 |
| g__norank_f__norank_o__SepB_3 | HV | 2.77 | 0.02 |
| g__Hydrogenophaga | HV | 2.76 | 0.01 |
| g__Defluviicoccus | HV | 2.75 | 0.01 |
| g__unclassified_f__Sphingomonadaceae | HV | 2.73 | 0.01 |
| g__Alsobacter | HV | 2.7 | 0.01 |
| g__norank_f__cvE6 | HV | 2.7 | 0.01 |
| g__norank_ c__Verrucomicrobiae | HV | 2.7 | 0.01 |
| g__Clostridium_sensu_stricto_13 | HV | 2.69 | 0.01 |
| g__Vogesella | HV | 2.69 | 0.01 |
| g__unclassified_c__Gammaproteobacteria | HV | 2.68 | 0.01 |
| g__norank_f__Lentimicrobiaceae | HV | 2.66 | 0.01 |
| g__norank_f__Vermiphilaceae | HV | 2.65 | 0.01 |
| g__norank_f__0319_6G20 | HV | 2.64 | 0.01 |
| g__Paenibacillus | HV | 2.63 | 0.01 |
| g__GKS98_freshwater_group | HV | 2.62 | 0.01 |
| g__norank_f__67_14 | HV | 2.56 | 0.01 |
| g__Epulopiscium | HV | 2.54 | 0.01 |
| g__norank_f__Prolixibacteraceae | HV | 2.53 | 0.01 |
| g__Agathobacter | HV | 2.51 | 0.02 |
| g__Lacunisphaera | HV | 2.49 | 0.01 |
| g__unclassified_c__Deltaproteobacteria | HV | 2.49 | 0.01 |
| g__norank_f__Sandaracinaceae | HV | 2.46 | 0.02 |
| g__Luteitalea | HV | 2.42 | 0.02 |
| g__Mesorhizobium | HV | 2.41 | 0.01 |
| g__Ferruginibacter | HV | 2.4 | 0.01 |
| g__norank_f__Gemmataceae | HV | 2.4 | 0.01 |
| g__Acidovorax | HV | 2.39 | 0.02 |
| g__Gleocapsa | HV | 2.38 | 0.01 |
| g__Clostridium_sensu_stricto_8 | HV | 2.37 | 0.01 |
| g__norank_ o__Gastranaerophilales | HV | 2.37 | 0.02 |
| g__Acetobacteroides | HV | 2.36 | 0.02 |
| g__norank_f__norank_o__EV818SWSAP88 | HV | 2.34 | 0.02 |
| g__norank_f__norank_o__Microtrichales | HV | 2.33 | 0.01 |
| g__Clostridium_sensu_stricto_1 | HV | 2.33 | 0.01 |
| g__norank_f__norank_o__R7C24 | HV | 2.3 | 0.01 |
| g__Candidatus_Paracaedibacter | HV | 2.29 | 0.01 |
| g__unclassified_c__Subgroup_6 | HV | 2.28 | 0.01 |
| g__unclassified_f__Propionibacteriaceae | HV | 2.28 | 0.01 |
| g__unclassified_f__Acetobacteraceae | HV | 2.27 | 0.01 |
| g__Fodinicola | HV | 2.23 | 0.02 |
| g__unclassified_o__Chlamydiales | HV | 2.23 | 0.01 |
| g__norank_f__Caldilineaceae | HV | 2.22 | 0.01 |
| g__Micromonospora | HV | 2.22 | 0.01 |
| g__norank_f__Rubritaleaceae | HV | 2.22 | 0.01 |
| g__norank_f__norank_o__norank_c__Alphaproteobacteria | HV | 2.19 | 0.01 |
| g__Woeseia | HV | 2.19 | 0.02 |
| g__Coxiella | HV | 2.18 | 0.01 |
| g__Methylobacterium | HV | 2.16 | 0.01 |
| g__norank_f__norank_o__CCM19a | HV | 2.14 | 0.02 |
| g__LD29 | HV | 2.11 | 0.01 |
| g__Paucibacter | HV | 2.11 | 0.02 |
| g__norank_f__Steroidobacteraceae | HV | 2.08 | 0.01 |
| g__Nakamurella | HV | 2.04 | 0.01 |
| g__norank_f__Desulfarculaceae | HV | 2.01 | 0.02 |
