# Supplementary material for: Gut bacterial communities in the freshwater snail Planorbella trivolvis and their modification by a non-herbivorous diet
Source: PeerJ. 2021 Feb 12;9:e10716. doi: 10.7717/peerj.10716 (PMC7883694; doi:10.7717/peerj.10716)
Supplement: Supplemental Information 3 [file peerj-09-10716-s003.docx]

The diameter of shell of the snails (mm)

|  |  |  | Repetition | | |  |  |
| --- | --- | --- | --- | --- | --- | --- | --- |
|  |  |  | 1 | 2 | 3 | Mean | SD |
| HV | Initial value | | 5.91 | 5.98 | 6.05 | 5.98 | 0.07 |
|  | Final value |  | 10.54 | 10.29 | 10.76 | 10.53 | 0.24 |
| NHV | Initial value | | 5.65 | 5.91 | 5.79 | 5.78 | 0.13 |
|  | Final value |  | 11.76 | 11.85 | 11.98 | 11.86 | 0.11 |

The weigt of the snails (g)

|  |  |  | Repetition | | |  |  |
| --- | --- | --- | --- | --- | --- | --- | --- |
|  |  |  | 1 | 2 | 3 | Mean | SD |
| HV | Initial value | | 0.069 | 0.064 | 0.071 | 0.068 | 0.004 |
|  | Final value | | 0.216 | 0.22 | 0.221 | 0.219 | 0.003 |
| NHV | Initial value | | 0.064 | 0.062 | 0.072 | 0.066 | 0.005 |
|  | Final value | | 0.431 | 0.425 | 0.439 | 0.432 | 0.007 |
