# Supplementary material for: Gut bacterial communities in the freshwater snail Planorbella trivolvis and their modification by a non-herbivorous diet
Source: PeerJ. 2021 Feb 12;9:e10716. doi: 10.7717/peerj.10716 (PMC7883694; doi:10.7717/peerj.10716)
Supplement: Supplemental Information 4 [file peerj-09-10716-s004.docx]

Nutritional ingredient of the diets

| Diets type |  | Protein | Crude fiber | Crude fat | Crude ash |
| --- | --- | --- | --- | --- | --- |
| HV(alfalfa) | 1 | 19.41 | 26.86 | 4.75 | 8.36 |
|  | 2 | 19.59 | 26.47 | 4.86 | 8.35 |
|  | 3 | 19.87 | 26.91 | 4.97 | 8.32 |
|  | Mean | 19.62 | 26.75 | 4.86 | 8.34 |
|  | SD | 0.23 | 0.24 | 0.11 | 0.02 |
| NHV(pellet feed) | 1 | 27.13 | 4.95 | 2.12 | 9.04 |
|  | 2 | 27.31 | 4.96 | 2.21 | 8.97 |
|  | 3 | 27.22 | 5.01 | 2.18 | 9.09 |
|  | Mean | 27.22 | 4.97 | 2.17 | 9.03 |
|  | SD | 0.09 | 0.03 | 0.05 | 0.06 |
