# Supplementary material for: Gut bacterial communities in the freshwater snail Planorbella trivolvis and their modification by a non-herbivorous diet
Source: PeerJ. 2021 Feb 12;9:e10716. doi: 10.7717/peerj.10716 (PMC7883694; doi:10.7717/peerj.10716)
Supplement: Supplemental Information 5 [file peerj-09-10716-s005.docx]

| Statistical **Analysis** | **R package and other program** |
| --- | --- |
| PCoA | vegan package in R |
| Venn | vegan package in R |
| Heatmap | Heatmap package in R |
| KO functions | PICRUSt |
